# Evidence of Habitat Structuring *Aedes albopictus* Populations in Réunion Island

**DOI:** 10.1371/journal.pntd.0002111

**Published:** 2013-03-21

**Authors:** Hélène Delatte, Céline Toty, Sébastien Boyer, Anthony Bouetard, Fanny Bastien, Didier Fontenille

**Affiliations:** 1 Maladies Infectieuses et Vecteurs: Ecologie, Génétique, Evolution et Contrôle, Institut de Recherche pour le Développement 224, Centre National de la Recherche Scientifique 5290, Universités Montpellier 1 & 2, Montpellier, France; 2 CIRAD, UMR PVBMT, F-97410 Saint-Pierre, La Réunion, France; 3 Centre de Recherche et de Veille sur les Maladies Emergentes dans l'Océan Indien, Sainte Clotilde, Réunion Island, France; University of California, Davis, United States of America

## Abstract

Arbovirus vector dynamics and spread are influenced by climatic, environmental and geographic factors. Major Chikungunya and Dengue fever outbreaks occurring the last 10 years have coincided with the expansion of the mosquito vector *Aedes albopictus* to nearly all the continents. We characterized the ecological (larval development sites, population dynamics, insemination and daily survival rates) and genetic (diversity, gene flow, population structure) features of two *Aedes albopictus* populations from distinct environments (rural and urban) on Réunion Island, in the South-West Indian Ocean. Microsatellite analysis suggests population sub-structuring *Ae. albopictus* populations. Two genetic clusters were identified that were significantly linked to natural versus urban habitats with a mixed population in both areas. *Ae. albopictus* individuals prefer urban areas for mating and immature development, where hosts and containers that serve as larval development sites are readily available and support high population densities, whereas natural environments appear to serve as reservoirs for the mosquito.

## Author Summary

The objective of our research was to study the movements the mosquito *Aedes albopictus*. This mosquito transmits more than 20 viruses to humans throughout the world and is the vector of the recent major epidemics of Dengue and Chikungunya on Reunion Island and the Indian Ocean Region and is, therefore, of great interest for human health. We set out to determine whether reservoirs of populations could be found in natural environments and whether or not these populations are capable of re-colonising urban areas. Until now, only limited data has been available on the population dynamics of *Aedes albopictus* in this part of the world, information critical for guiding vector control strategies and predicting or preventing epidemics. We chose two areas where a serious CHIKV epidemic occurred. We then used genetic markers and ecological data to estimate patterns of gene flow and behaviour. We were able to demonstrate that populations were structured with limited gene flow despite observing migration. We found that *Ae. albopictus* preferred urban areas for mating and to lay their eggs because of the availability of hosts and permanent containers that favoured higher mosquito densities. We also show, however, that natural environments are reservoirs for re-colonisation of urban areas.

## Introduction

In recent years, the emergence of arboviruses and some of their vectors has caused major health and economic problems worldwide. Chikungunya (CHIK), an arbovirus infection that was not considered to be a major health problem before 2005, recently caused a major pandemic affecting Africa, Asia and to a lesser extent Europe. The pandemic began in Kenya and the South-West Indian Ocean in 2005 [1], [2], with a separate focus in Central Africa [3], [4] that then spread to Europe [5] and Asia [6]. Thousands of people were affected with incidence rates up to 75% in Lamu, Kenya [7]. In addition to low levels of immunity against CHIV in the human population, emergence of epidemic transmission has been attributed to changes in vector competence [8], ecology [9], [10] and dynamics [11]. It is hypothesized that an amino acid replacement in the E1 envelope glycoprotein arose in response to selection for efficient transmission by *Aedes albopictus* especially in locations where *Ae. aegypti* was absent or less abundant [8], [12], [13] . Therefore, the vectors incriminated for this pandemic were primarily *Ae. albopictus* and to a lesser extent *Ae. aegypti* [14], [15]. *Aedes albopictus* originated in Asia [16] and has extended its range in the last 20 years across many parts of the world. It is now recognized as a competent vector of numerous arboviruses [14], [17].

*Aedes* vector dynamics and spread are affected by climatic [18], [19], environmental and geographic factors [20], [21]. These vector species are known to be short-distance migrants and their dynamics are influenced by their environment [22]. The flight ranges of *Aedes albopictus* may increase when females fail to find a suitable site for oviposition or blood-meals. Its abundance varies from year to year and is affected by the inter-annual climate variability [23]. Indeed, understanding the factors that determine the vectors' habitat and population dynamics at a micro-scale is a major challenge but could help improve the efficiency of vector control.

Réunion Island is situated in the South-West Indian Ocean, East of Madagascar. The habitat is predominantly composed of houses with gardens and more than 300 gullies spread throughout the island. The gullies cross urban environments and natural areas, providing potential mosquito production sites. Nevertheless, this habitat has never been evaluated for its impact on human health as a potential reservoir for mosquitoes, especially *Ae. albopictus*, the dominant species on the island [15]. Indeed, the population densities, dynamics or flow between the gullies and the urban environments have never been investigated. In this study, we seek to examine the population ecology (larval development sites, longevity and insemination rates) and genetic structure during two seasons in two locations, including gullies and urban areas.

## Materials and Methods

### Statement of Ethics

All volunteers are co-authors and provided informed oral consent as the IRB approved the use of oral consent. Oral consent was obtained before starting the whole study, after clear explanation of what would stand in the study. All entomological surveys and gathering made on private lands or in private residences were made with the owners/residents permission and presence.

### Study Area

We chose two gullies (approximately 300 meters long) close to an urbanised area bordering the gully (13 houses were surveyed): one situated on the eastern part (Chemin Sévère, close to the main city Saint Benoît), the other on the western side of the island (Bassin Plat, close to the main city Saint Pierre). The distance between the gullies and houses was less than 20 meters. Both sites are infested with *Ae. albopictus*; in 2007 the average Breteau Index was 38 in Saint-Benoît and 28 in Saint-Pierre (Agence Régionale Santé [ARS]) and had a high number of CHIKV cases reported in 2005–2006 (*Bulletin Épidémiologique Hebdomadaire*, Institut de Veille Sanitaire, N° 38-39-40, 21 October 2008). Seroprevalences of antibodies against CHIKV estimated after the epidemic were 48% and 38% in the Saint Benoît and Saint Pierre, respectively [24].

Houses surveyed were built in concrete and/or wood and tin- roofed. Houses had, as in most urban areas on La Réunion, a large grass-garden combined with numerous varieties of fruit trees, flower beds, bushes and other flowering shrubs.

### Entomological Surveys

Entomological surveys were conducted 1 gully and 13 nearby houses in both the Chemin Severe (East) and Bassin Plat (West) sites the austral winter in 2006 (July/August) and summer in 2007 (February/March). Container surveys for immature mosquitoes and human landing collections for adult mosquitoes were carried out on the same day at each site. Adult samplings were performed under a shaded environment (for example in the gully in the east that was under the shade of a bamboo grove, in the west under shade of *Schinus therebenthifolius* grove, in urban parts under the shade of fruit trees). At each season about 2 weeks were needed for each site to perform all entomological surveys, larvae and adult collections.

#### Immature surveys

Both gullies and residential properties were inspected for water holding containers and naturally occurring larval habitats. Natural habitats included bamboo stumps, and tree and rock holes which were generally found in gullies. The remaining containers were classified into the following categories: plates under flowerpots, big (1.5 to 10 L) and small containers (<1.5 L), tyres, basins and tanks and *bromeliaceae* in gardens (Table 1). For each wet container identified, the depth of the water was measured, and then emptied into a separate container to measure the volume. Each container was scored subjectively for organic content of the water (low, medium, high), shade (no direct sunlight, exposed to sunshine at least once during the day), and water quality (clear = colorless, tinted = in between, polluted = opaque and stinky).

**Table 1:** Typology of *Aedes albopictus* production sites.

Season	Habitat	Location	Site class	No. positive	Total Aedes		Aedes/positive		Mean Width	Mean Depth	Positive production	
							production sites		(cm)	(cm)	sites containing	
				/No. inspected	Larvae	Pupae	Larvae	Pupae			Culex	Anopheles
**Winter**	**Gully**	**East**	0	140/145 (97%)	1048	176	7.49	1.26	6,41±4,05	6,70±5,72	0	1
			1	2/4 (50%)	30	12	15.00	6.00	20,25±4,92	6,00±2,31	2	0
			2	0/5 (0%)	0	0	0.00	0.00	7,00±1,00	9,20±13,33	0	0
			4	2/3 (67%)	110	10	55.00	5.00	30.00	5,50±3,12	1	0
**Winter**	**Gully**	**West**	0	12/12 (100%)	448	114	37.33	9.50	21,58±9,80	4,25±4,08	2	0
			1	1/1 (100%)	1	2	1.00	2.00	20.00	3.00	0	0
			4	2/2 (100%)	52	10	26.00	5.00	13,5±16,26	2,60±0,57	0	0
			Other	1/1 (100%)	39	9	39.00	9.00	15.00	6.00	0	0
**Winter**	**Houses**	**East**	0	7/10 (70%)	234	87	33.43	12.43	4,62±1,60	3,25±3,06	1	0
			1	53/88 (60%)	1247	225	23.53	4.25	18,24±6,75	4,46±4,87	1	0
			2	12/21 (57%)	128	10	10.67	0.83	3,50±1,87	7,93±7,31	0	0
			3	9/18 (50%)	117	22	13.00	2.44		16,89±23,54	2	0
			4	3/9 (33%)	122	31	40.67	10.33	25.00	6,74±2,49	2	0
			5	14/21 (67%)	466	82	33.29	5.86	42,44±48,90	11,80±21,35	2	0
			6	15/27 (56%)	105	4	7.00	0.27	8,53±12,50	6,59±4,13	1	0
			Other	5/11 (45%)	165	27	33.00	5.40	2,5±0,00	17,59±27,26	0	0
**Winter**	**Houses**	**West**	0	1/4 (25%)	112	0	112.00	0.00		15,25±13,30	1	2
			1	5/8 (63%)	72	4	14.40	0.80	24,5±0,71	2,81±2,75	3	0
			2	0/1 (0%)	0	0	0.00	0.00		11.00	0	0
			3	3/3 (100%)	362	76	120.67	25.33	27±13,45	5,33±2,89	1	0
			5	1/3 (33%)	118	2	118.00	2.00		19,67±17,79	1	0
			6	0/1 (0%)	0	0	0.00	0.00		0.50	1	0
			Other	0/8 (0%)	0	0	0.00	0.00	5.00	5.00	7	0
**Summer**	**Gully**	**East**	0	119/196 (61%)	1260	121	10.59	1.02	8,67±8,23	4,83±4,86	33	5
			4	0/1 (0%)	0	0	0.00	0.00	15.00	6.00	1	0
**Summer**	**Gully**	**West**	0	7/22 (32%)	320	25	45.71	3.57	41,05±29,16	5,29±5,50	4	0
			1	1/1 (100%)	55	2	55.00	2.00	18.00	3.00	0	0
			4	2/2 (100%)	68	3	34.00	1.50	30±0,00	2,60±3,39	0	0
			5	1/1 (100%)	44	0	44.00	0.00	23.00	2.00	0	0
**Summer**	**Houses**	**East**	0	0/1 (0%)	0	0	0.00	0.00		1.00	0	0
			1	11/16 (69%)	1141	103	103.73	9.36	22,50±7,21	10,12±24,57	0	0
			2	2/6 (33%)	23	6	11.50	3.00	12,00±7,38	7,25±6,75	1	0
			3	8/10 (80%)	291	78	36.38	9.75	21,53±11,72	7,74±9,09	1	0
			4	4/7 (57%)	53	24	13.25	6.00	17,33±8,48	6,71±5,38	4	0
			5	7/12 (58%)	746	114	106.57	16.29	32,35±18,29	16,36±12,62	6	0
			6	12/15 (80%)	73	10	6.08	0.83	4,36±2,10	4,08±5,68	0	0
			Other	3/4 (75%)	121	20	40.33	6.67	22,00±10,74	5,08±6,68	1	0
**Summer**	**Houses**	**West**	0	3/3 (100%)	0	0	0.00	0.00	1,00±0,00	1,00±0,00	0	0
			1	13/14 (93%)	266	32	20.46	2.46	20,86±9,69	3,90±6,24	0	0
			3	1/1 (100%)	24	0	24.00	0.00	16.00	5.00	0	0
			4	3/4 (75%)	503	66	167.67	22.00	35,00±20,30	6,25±3,80	0	0
			6	4/6 (67%)	57	0	14.25	0.00		3,17±0,98	0	0
**Total**				489/728 (67%)	10021	1507	20.49	3.08	13.63±0.70	6.48±0.42	79	8

Distribution of the total number of pupae and larvae sampled in the field in two different locations (east/west), during two different seasons (winter/summer) in two different habitats (gully/urban). Site classes were as follow: 0: Natural, 1: Flowerpot plates, 2: Small containers, 3: Big containers, 4: Tyres, 5: Basin and tank and 6: Bromeliad plants. To be noticed, that no mosquitoes were collected from the western site from natural developmental sites in the urban habitat during the winter or from artificial developmental sites in the gully habitat during the summer.

Any larvae and pupae were collected using a pipette, counted and transported to the lab where they were reared until emergence for species identification. All *Ae. albopictus* that emerged were pooled by site (East/West), season (winter/summer), habitat (gully/urban) and type of developmental site (artificial/natural). Each pool of mosquitoes was preserved in alcohol (95%) and stored at −20°C for genetic analysis.

#### Human landing collections

Adult mosquitoes were collected as they landed on two human volunteers with a mouth aspirator before biting on exposed skin. Each site and habitat was sampled during each season (Table 1). Mosquitoes were collected until 120 adult female mosquitoes were obtained per site/habitat/season combination; because adult densities were high required approximately 1 hour with two volunteers. All mosquitoes were placed on ice and transported the laboratory.

### Parity Rate Determination and Spermathecae Dissection

Females *Ae. albopictus* were dissected to determine parity and the number of spermathecal capsules filled. Once the parity status [25] of each female had been determined, the three spermathecal capsules were placed in a drop of saline water on a glass slide covered with a glass cover slip and examined for sperm under a microscope.

### DNA Extraction

Each mosquito was ground in 200 µl of 2% CTAB with a glass bid using a Mix Miller MM 400 set at 30 Hz and left for 5 min at 65°C. Then, 200 µl of chloroform was added and mixed gently. After a centrifugation (12 000 rpm, 5 min), the upper phase was collected and 200 µl of isopropanol added. The mix was centrifuged for 15 min (12 000 rpm) and the isopropanol was removed. Then, an extra step of 70% ethanol was carried out to purify the DNA. After the removal of the 70% ethanol, the DNA was dried using a speed-vac and eluted with 20 µl of water.

### Choice of Microsatellite Markers and Microsatellite Library

Of the six primers available from the literature for *Ae. albopictus* [26], one (AealbB52, Table 2) was not variable and the genetic resolution obtained using the remaining five markers was not considered adequate. We screened some *Ae. aegypti* microsatellite markers available from the literature and selected two new markers (AEDC and 34–72, Table 2). In parallel we developed an enriched microsatellite bank, from which we identified two additional markers (see Table 2).

**Table 2:** Microsatellite sequences, accession numbers, repeat motives, size ranges and references.

Locus names	Accession numbers	Repeat motive	Forward sequence	Reverse sequence	Range	Reference
**AealbB52**	DQ366024	(AC)A(AC)A(AC)2 … (AC)6 … (T)3G(T)5G(T)4GGG(AC)3	GGGTCTAGAAGTAATAGCGATG	GCATTCTTTGCTTCTGTTTGC	173	Porreta *et al.*, 2006
**AealbB51**	DQ366023	(AC)3T(AC)2AA(AC)AAA(AC)3AA(AC)AT(AC)2T(AC)2	5′VIC-TCCACGTGGTATAACTCTGA	GTAGTTGTCCAATTAACATCG	124–167	Porreta *et al.*, 2006
**AealbA9**	DQ366022	(AC)4GCAT(AC)2TC(AC)8CCAA(AC)2CG(AC)GT(AC)C(AC)AT(AC)	5′PET-TGGGACAAGAGCTGAAGGAT	CTCGTTCTCTACTCTCTCCGTT	142–162	Porreta *et al.*, 2006
**AealbB6**	DQ366026	(AC)1AT(AC)7GC(AC)2GCAT(AC)6AG(AC)	5′HEX-ATGAGGTGACCCTTTTGTGC	AAATTTTATAGGGCCCTCGG	128–162	Porreta *et al.*, 2006
**AealbD2**	DQ366021	(A)16(AC)9GC(AC)22	5′VIC-GAATCCCACACAGCGTCTTT	GGTCGCTTGACACCTTGAAT	181–216	Porreta et *al.*, 2006
**AealbF3**	DQ366027	(AC)6AT(AC)3AAAA(GC)2	5′HEX-CTCGTGAGTACGTTCCGTGA	AGGGAAACAAGGACTTCATCA	215–245	Porreta *et al.*, 2006
**AEDC**	T58313	(GTA)6(ACG)(GTA)3	5′FAM-TGCAGGCCCAGATGCACAGCC	TCCGCTGCCGTTGGCGTGAAC	210–230	Chambers *et al.* 2007
**34–72**	AF338656	GAAAA(GA)6CAGACAGGAAA	5′FAM-CGTAGTGATTCTGTGATA	TGGCATCAGATTCAGTAA	178–204	Huber *et al.* 2001
**alb212**	JQ886085	(AC)5 (AC)6	GGAGTGTCCTCTCACCATC	GACGAACTGAGCAAATGTCT	145–245	This study
**alb222**	JQ886086	(GT)6	GACGAGAACGGTGAACAG	GTCGAAGGTACAAATAGATCG	209–239	This study

### Microsatellite Amplification and Genotyping

The extracted DNA of each sample was used as a template for the amplification of a set of 10 microsatellite markers AealbB52, AealbB51, AealbA9, AealbB6, AealbD2, AealbF3, alb212, 34–72, AEDC, alb222 (Table 2). These markers were selected for polymorphism, size, and low numbers of null alleles. Two were from the newly developed set, eight were from *Ae. albopictus* (6) and *Ae. aegypti* (2) literature (Table 2). A total of 342 adults were genotyped with these markers (Table 3). Genomic (10 ng) DNA was used for amplification with the QIAGEN multiplex PCR Master Mix kit (ref. 206145) according to the manufacturer's instructions in a final volume of 15 µL. One of each pair of primers was fluorescently end-labelled with the fluorochromes NED, VIC, PET or FAM. Two primer mixes were used in 15 µL at a final concentration of 400 nM. The programme consisted of denaturation at 94°C for 5 min followed by 30 cycles at 94°C for 45 s, 56°C for 1 min 30 s, 72°C for 45 s, with a final elongation step for 30 min at 60°C. Then, 2 µL of the DNA was diluted from 1/100 to 1/60 according to PCR products. The diluted PCR product was mixed with 10.7 µL of ultra-pure Hi-Di-formamide TM and 0.3 µl of size marker (GeneScan 500Liz), and loaded onto an ABI Prism 3130 Genetic Analyser automated sequencer. Allele sizes were determined using GeneMapper v4.0.

**Table 3:** A*edes albopictus* larvae sampling for the genetic analysis.

	Season		Habitat		Production sites	
East (128)	Winter	38	Gully	68	Natural	90
	Summer	90	Urban	60	Artificial	38
West (203)	Winter	88	Gully	118	Natural	90
	Summer	115	Urban	85	Artificial	113

### Genetic Analysis

#### Diversity analysis

Microsatellite diversity within populations was estimated using observed (Ho) and Nei's 1987 unbiased expected heterozygosity (He) in Genetix 4.03 [27]. All pairs of loci were tested for linkage disequilibrium using the probability test in Genepop [28]. Single and multilocus Fis were estimated using Weir & Cockerham's fixation index (1984). Deviations from the Hardy–Weinberg equilibrium (HWE) were tested using a two-tailed Fisher's exact test based on Markov-chain randomisation (1000 dememorisations, 100 batches and 1000 iterations per batch) in Genepop.

#### Population differentiation and structure

Population differentiation was quantified by calculating pairwise Fst values [29]. Significance was verified using the permutational genetic discontinuities among clusters and areas/habitats (east/west and gully/urban) and quantified using the hierarchical analysis of molecular variance (AMOVA). Clusters were grouped according to different combinations: Structure clusters, season, sites (East/West), habitat (gully/urban) and type of larval habitat (natural/artificial) (see results). Differences in the partition of genetic variation (Fct) among and within (Fis) regions were tested using nonparametric permutational procedures (1,023 iterations) of Arlequin 3.5 [30]. Levels of population admixture were quantified using a number of Bayesian clustering procedures as implemented in Structure 2.3.3 [31]. Structure can be used to calculate clustering patterns based on multilocus genotypes and makes it possible to correct for the presence of null alleles [32]. Analyses in Structure were based on the admixture model with no prior information about the population. In order to allow asymmetric patterns of admixture amongst populations, the Dirichlet parameter for degree of admixture (a) was separately determined for each population [31]. The number of population clusters was determined according to Evanno *et al.* [33]. The ad hoc DK statistic was calculated for K ranging from 1 to 10. Structure was run for 10 million generations (burn-in = 100,000 generations) with 10 iterations for each value of K. To use structure, HWE and linkage equilibrium are assumed for each group. Both hypotheses were tested a posteriori on each cluster.

### Statistical Analysis

A mixed-data factor analysis was carried out on our datasets containing a combination of continuous and ordinal variables. The resulting components were used in regression models and tested with an ANOVA. A step-by-step analysis was done with all the different factors, removing the less significant factor at each step. At each step, we compared the tested model and the previous model until a significant difference appears between the two models. The final retained model was the model before this significant difference appears. We used a model with multivariate normal random effects, using Penalized Quasi-Likelihood. This general linear model is used to fit generalized linear models, specified by giving a symbolic description of the linear predictor and a description of the error distribution. Specifically, the GLMM is assumed to be of the form g(μ) = Xβ+Ze where g is the link function, μ is the vector of means and X, Z are design matrices for the fixed effects β and random effects e respectively. Furthermore the random effects are assumed to be i.i.d. N(0, σ2). The 14 studied factors are the width, depth, volume, class and type of the breeding site, type of habitat, the location, organic matter, effect of the sun, the season, the year and the presence of Anopheles and Culex and the type of water quality. The year was considered as a randomised factor. The models and the ANOVA were carried out on a dataset from 728 immature production sites. All statistical analyses of this part were performed with R software.

#### Parity analysis

Differences in the proportion of parous females among different habitats (urban/gully), seasons (winter/summer), and location (east/west) was tested by Chi-square. The influence of the location, the habitat and the seasonal factors on the number of filled spermathecae was tested with an ANOVA. For location on the number differences in the number of filled spermathecae (0, 1, 2 or 3 filled spermatheacae) was tested by Chi-square. The effect of habitat on the distribution of *Ae. albopictus* in the different population clusters was tested using a Chi-square test. The differences between the habitats related to each cluster determined with the microsatellites analyses were tested with Tukey tests. All statistical analyses of this part were carried out using JMP 8.0 software.

#### Daily survival rate

The daily survival rate (p) was calculated using the parity rate (M) and the time of the first gonotrophic cycle (i0) using the formula p = M∧(1/i0) [34], [35]. The length of the gonotrophic cycle has been estimated with the results obtained with the same *Aedes albopictus* population in a study carried out by Delatte et al., [36].

## Results

### Ecological Characterisation of the *Aedes albopictus* Habitat

A total of 11,528 *Ae. albopicutus* larvae and pupae were collected from 728 potential larval development sites (Table 1). Of these, figure the 8,634 immature individuals collected from 630 containers or natural habitats located on the east side of the island compared to 2, 890 collected in 99 containers on the west side of island. Abundance of larval habitats were comparable during the winter and summer collections (Table 1). Although, slightly more potential larval habitats were observed in gullies compared to household collections (396 versus 333), significantly more larvae and pupae were collected in houses (nearly 2 fold 7,565 versus 3,959). The average number of immature *Ae. albopictus* ranged from: 8 to 13 in natural production sites and small containers; 37 to 46 in plates under flowerpots, tyres and big containers; and 82 in basins and tanks.

Six of the 14 factors tested (see material and method section), significantly influenced the number of immature mosquitoes present in the production sites (Table 4). The width, volume and nature of the breeding site were correlated with the number of immature *Ae. albopictus*, as well as the mosquito's habitat and the location. The number of Aedes individuals was significantly higher in the biggest and widest breeding sites. We observed significant differences between the average numbers of immature *Ae. albopictus* (+/− SE) from natural and plant immature production sites (13.10±2.58) compared to the artificial immature production sites (42.65±3.54). The total number of immature mosquitoes was higher in the east than in the west. However, average productivity was significantly higher in the west (47.31±6.07) than in the east (19.96±2.29). A significant difference was observed between the number of immature mosquitoes from gullies or urban areas. On average, *Ae. albopictus* immature production site productivity was 13.53±3.32 in gullies and 37.53±3.32 in urban areas. The number of immature *Ae. albopictus* was not correlated to the presence of sun (yes/no), water quality (clear/tinted/polluted), the presence of organic matter, the season (winter/summer) and the presence of *Anopheles and Culex*.

**Table 4:** Factors influencing the number of immature *Aedes albopictus* (pupae and larvae, n = 11528) issued from the sampling of 728 breeding sites.

	numDF	denDF	F-value	P-value	
**(Intercept)**	1	143	1748.38	<.0001	***
**Landscape**	1	143	13.08	0.0004	***
**Season**	1	143	0.83	0.3642	N.S.
**Type of Habitat**	1	143	20.79	<.0001	***
**Class of Production site**	6	143	9.22	<.0001	***
**Production site depth**	1	143	2.33	0.1294	N.S.
**Production site volume**	1	143	5.01	0.0267	*
**Production site width**	1	143	44.04	<.0001	***
**Sunshine**	1	143	0.11	0.7365	N.S.
**Water**	1	143	0.27	0.6037	N.S.
**Organic matter**	2	143	2.01	0.1376	N.S.
**Density**	2	143	2.15	0.1201	N.S.
**Distance to habitat**	1	143	2.94	0.0886	N.S.
**Location**	1	143	17.02	0.0001	***

A step-by-step GLMM analysis was carried out on 14 factors (The table present the simplest model).p-value significance is represented by *, NS meaning “non-significant.”

### Parity and Insemination Rates

**Figure 1:**
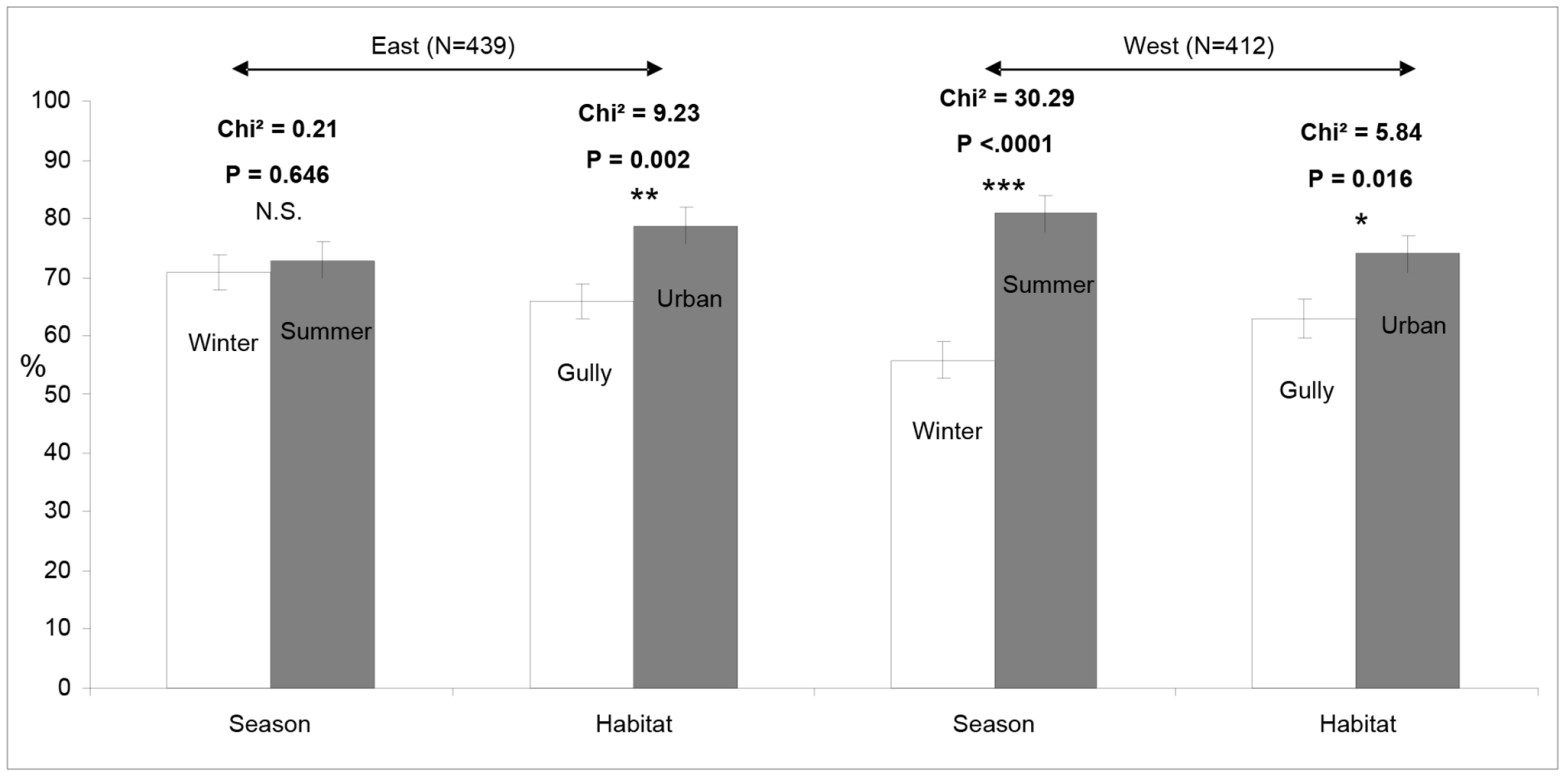
Parous rates of *Aedes albopictus* populations. Parous rate is presented according to their location (east/west), season (winter/summer) and habitat (gully/urban) sampled (n = 851). The difference of distribution was tested with a Chi-square test.

A total of 851 *Ae. albopictus* adult females were dissected to determine parity and the number of spermathecae that were inseminated. Overall 70.2% were parous (598/851). On both sides of the island, the proportion of parous females was higher in houses than in gullies (Figure 1). In contrast, the effect of season on parity differed between the two sides of the island; in the west parous rate in the summer was 56.1±1.9% compared to 81.0±2.1% during the winter (Figure 1) whereas in the eastern sites there was no significant difference between the parous rates during the two seasons. The daily survival rate was higher in urban areas and during the winter. Thus, younger mosquito populations were found in gullies (Table 5).

**Table 5:** Parity rates and daily survival rates on sampled populations.

	East		West		Season	
	Gully	Urban	Gully	Urban	Summer	Winter
**Parous rate**	0.646	0.765	0.630	0.740	0.561	0.810
**P with i0 = 3.5**	0.88	0.93	0.88	0.92	0.85	0.94

The daily survival rate (p) was calculated using the parous rate (M) and the time of the first gonotrophic cycle (i0) with the formula p = M∧(1/i0).

**Figure 2:**
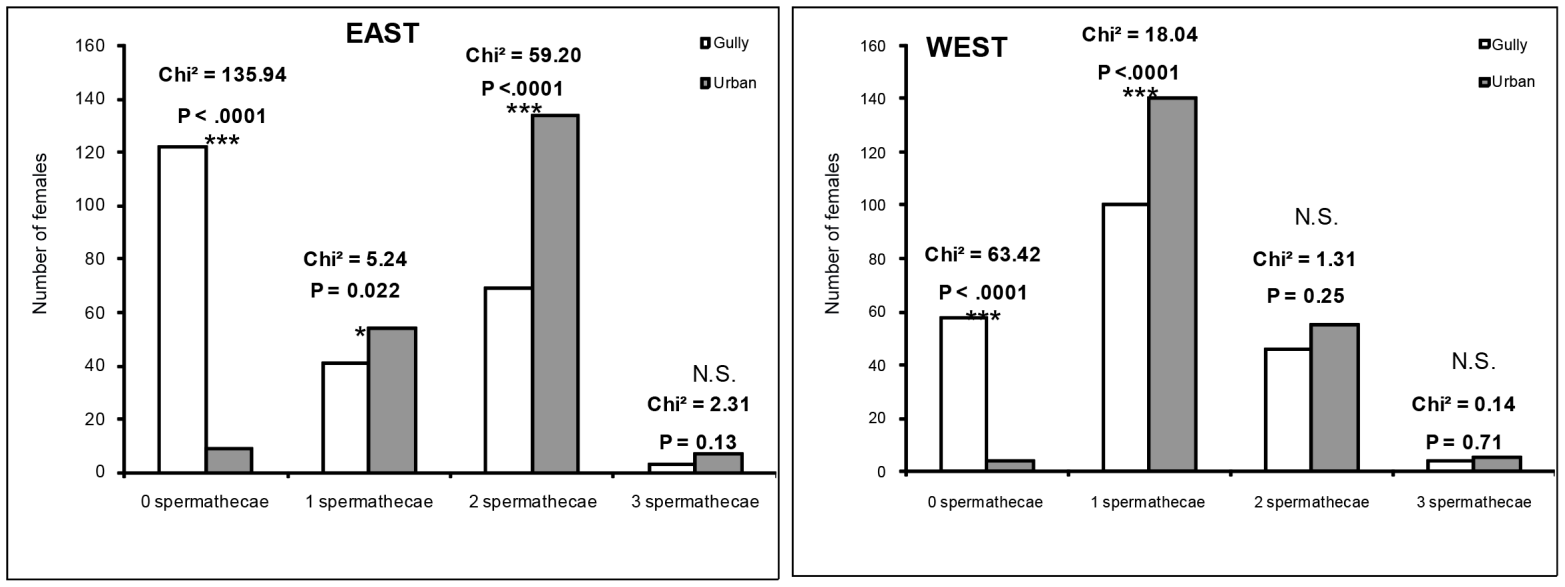
Number of female *Aedes albopictus* with spermathecal capsules status (n = 851) according to sampling sites.

The dissection of the spermathecal capsules of the same 851 adult females showed that 193 had empty spermathecae, 335 had one filled spermathecae, 304 had two filled spermathecae and 19 had three filled spermathecae (Figure 2). Independent of location, the number of filled spermathecal capsules in *Ae. albopictus* was higher in urban than gully habitats (ANOVA; Df = 1; F = 140.85; P<0.0001). On average, the number of empty spermathecae was significantly higher in the gully areas than in urban areas (Chi^2^, P<0.0001 Figure 2). There were significantly more females caught in urban areas with one full spermathecal capsule compared to females captured in the gully areas, independent of location (Figure 2). However, in the western location no significant differentiation was noticed between the different habitats for females with two filled spermathecae (Chi^2^, P = 0.25) unlike in the east (Chi^2^, P<0.0001).

### Genetic Diversity and Habitat

From a total of 342 mosquitoes collected, 11 produced no PCR products for fewer than six of the loci and were discarded (Table 3). The loci AealbB52 were monomorphic for our populations. The nine remaining loci had 4 to 17 alleles each, with allelic richness ranging from 4 to 13. On the 36 combinations of pairs of loci, only four combinations were in linkage disequilibrium.

Population structure among samples was investigated using assignation probabilities provided by Structure. Two groups (DK = 300) were identified of 139 and 114 mosquitoes, respectively (Figure S1). It was considered that an individual assignation probability in the [0.30; 0.70] interval belonged to a hybrid genotype and the others belonged to pure populations. A total of 78 hybrids were detected (Figure S2).

Significant deviation from Hardy-Weinberg Equilibrium was detected in seven markers (Table 6). The recorded deviations are likely due to null alleles because in each case there was an excess of homozygotes. The AMOVA showed that most of the variation was distributed within individuals (81%), but also between clusters identified in Structure (8.35%) (Table 7). The genetic differences between cluster 1 and cluster 2 account for more genetic variance (8.35%; Fct = 0.084, P<0.001) than those among habitats within clusters (1.5%; Fsc = 0.017, P<0.001). No significant differentiation was found among clusters between types of immature production sites (artificial/natural; data not shown) or season (summer/winter; data not shown).

**Table 6:** Allele frequency based correlation (*Fis*) and heterozygosity (H).

Populations	Allelic richness	He	Ho	Fis	Null allele frequency
**East summer urban artificial**	3.53	0.67	0.75	0.11*	0.13
**East summer urban natural**	3.52	0.70	0.73	0.04	0.11
**East winter gully natural**	3.24	0.53	0.68	0.22*	0.17
**East summer gully natural**	3.34	0.61	0.72	0.16*	0.14
**East winter gully artificial**	3.34	0.66	0.71	0.08	0.07
**West summer urban artificial**	3.62	0.65	0.75	0.14*	0.08
**West summer urban natural**	3.47	0.63	0.74	0.15*	0.11
**West summer gully artificial**	3.21	0.65	0.68	0.04	0.12
**West summer gully natural**	3.5	0.65	0.74	0.12*	0.12
**West winter urban artificial**	3.51	0.77	0.74	−0.04	0.08
**West winter gully artificial**	3.2	0.61	0.68	0.10*	0.12
**West winter gully natural**	3.38	0.68	0.71	0.04	0.12

Observed heterozygosity (HO), expected heterozygosity (He) and Weir and Cockerham's fixation index FIS (1984) were given by the software GenePop 4.0 and corrected using the Bonferroni test.

**Table 7:** Analysis of molecular variance (AMOVA).

Source of variation	Sum of squares	Percentage of variation	Fixation indices
**Among**			
**Groups (clusters 1&2)**	73.098	8.356	FCT: 0.084*
**Among populations (gully/urban)**			
**within groups**	16.022	1.531	FSC: 0.017*
**Among individuals**			
**within populations (gully/urban)**	772.864	9.346	FIT: 0.192*
**Within individuals**	656.000	80.766	FIS: 0.104*
**Total**	1517.985		

AMOVA and F-statistics of genetic differentiation between clusters 1 and 2 and among type of sampling sites (populations = gully/urban) of *Aedes albopictus* computed using the method proposed by Excoffier *et al.* 2005.

### Ecological Structuring of *Aedes albopictus* Populations

**Figure 3:**
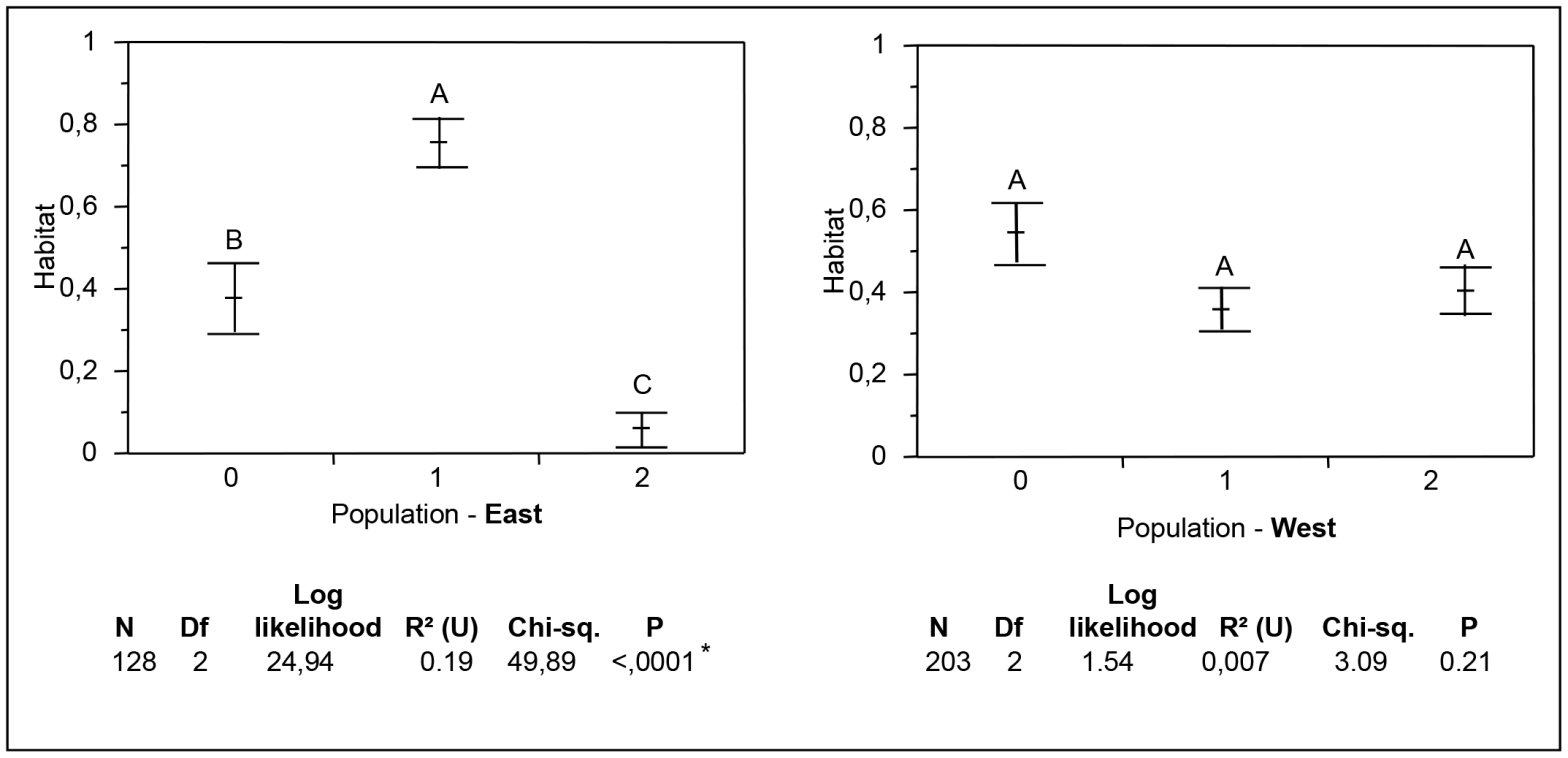
Distribution of *Aedes albopictus* clusters found with the genetic analysis according to habitat. Population clusters 0 to 2 have been assigned with Structure software, with cluster 0 being the hybrids between both clusters (with a threshold of 0.70). Chi-square tests were performed to determine the distribution of each cluster according to its habitat. Tukey tests were performed to compare the habitat belonging to each cluster.

The two clusters (plus a hybrid population) were assigned using Structure software, following the microsatellite analysis (see above). The 331 individuals analysed and assigned to one of the populations were studied in relation to their location (east/west), the season when they were sampled (summer/winter), their habitat (gully/urban) and the nature of the immature production sites (natural/artificial) (Table 5, 7, Figure S2). When the dataset was partitioned into the two locations sampled (east/west), we observed significant differences in the genetic distribution of these clusters according to their habitat (Figure 3). In the west, no difference was observed between the three clusters according to habitat. However, in the east, individuals from cluster 1 were significantly more present in the urban habitat and cluster 2 significantly in the gully habitat. Interestingly, the hybrid cluster was found in almost equal proportion in both gully and house habitats (Figure 3). No significant differentiation was found in terms of the nature of the immature production sites (artificial/natural).

## Discussion

There appeared to be two clusters of *Ae. albopictus* between which there is restricted gene flow. Despite the presence of these two genetic clusters in all the locations sampled, *Ae. albopictus* populations were structured in the eastern regions. In these regions, genetic clusters were significantly linked to habitat (gully or urban) with a mixed population that is present in both areas regardless of the type of larval development site or the season of sampling.

Significantly younger (nulliparous) and virgin (unmated) *Ae. albopictus* females were found in gullies compared to urban areas regardless of season and the side of the island sampled. This result suggests that the availability of human hosts and stable containers serving as larval development sites support large are very important factors for *Ae. albopictus* population dynamics. We hypothesize that gullies provide a larval development sites for mosquito populations, but that migration in search of hosts is likely to occur from gullies to urban areas. This is corroborated by the fact that the number of filled spermathecal capsules is higher overall in urban areas than in gullies. Similar results were found in experiments conducted on laboratory populations of *Ae. albopictus* under optimal conditions, where only 8% of the females had three filled spermathecal capsules [37]. The exchange of genes is supported by the study of the spermathecae, the parity and age, which showed a population movement from the gullies into urban areas.

The behaviour of the local zoophilic and anthropophilic *Ae. albopictus* [11], could explain the relative importance of the larval development sites compared to the availability of hosts, which are always present in gullies and urban areas. It is likely that given the high number of *Aedes* and the large number of hosts and their availability, vector control has caused a division between gully and urban clusters. These results differ from results for other *Aedes* species, such as *Ae. aegypti* populations in Cambodia, where patterns of differentiation between sympatric collections were associated with different container types [38]. *Ae. albopictus* was more abundant urban areas increasing the risk of virus transmission. The productivity of artificial immature production sites was much higher in urban areas than in gullies. This could be explained by the long-term availability of the former (anthropic immature production sites maintained by human activity, such as plates under flowerpots or containers for water storage) compared to natural sites. The existence of torrents in gullies, subjected to periodic flooding and drying, could explain why fewer *Aedes* were observed in those areas (but still with a high number observed, Table 1). In addition, there were fewer natural immature production sites and these are subject to frequent drainage because of the high rainfall. Nonetheless, natural environments in Réunion Island, such as gullies, should be considered as a potential risk for human health and as a nuisance, given the large *Ae. albopictus* population observed. The natural areas may not act as a barrier but could constitute a reservoir, particularly because they are available all year round (after anti-vectorial control, for example, which is largely targeted in urban areas). No restriction of gene flow was observed in the western region, while gene flow present in the eastern region it was restricted.

The differences between the eastern and western regions may be associated with climate; the eastern part of the island is very humid, with an average annual rainfall of 3,563 mm compared to the west, with an average of 1,030 mm rain/year (Météo France, 2005). Therefore, in the east there are more suitable larval habitats (Table 1). In contrast, on the west side of the island where suitable larval development sites are scarce, *Ae. albopictus* are more likely to migrate in search of suitable oviposition sites. This leads to an increase in the gene flow and is advantageous for population panmixia. Thus, dispersal of *Ae. albopictus* appears, in part to be driven by the availability of oviposition sites. This is demonstrated by the ability of *Ae. albopictus* to re-colonise neighbourhoods rapidly after environmental sanitation operations [39]. Containers that are near to other larval habitats are more likely to be productive and have a higher number of pupae than areas where larval habitats are scarce, as has been demonstrated for *Aedes aegypti* [40]. The isolation of potential oviposition sites reduced the likelihood that they would contain pupae and reduced the average number of pupae per container [40]. Furthermore, skip oviposition, where the females prefer laying eggs in multiple water collection [41], has been observed in *Aedes* species [42], thus enhancing population migrations when immature production sites are scarce.

Production of immature *Aedes albopictus* were correlated with the abundance of mosquito-positive containers. In most cases, the population density of the species is associated with the number of discarded containers in the habitat [43]. In Cambodia, similar results were obtained for habitat segregation (linked to levels of urbanisation), where authors found a habitat that genetically structured *Ae. aegypti* populations [44]. In Peru, *Ae. aegypti* were spatially clustered indicating limited dispersal between households [45]. This has also been shown between species of mosquitoes. In Florida, for example, habitat segregation has been observed according to habitat variables associated with urbanisation and rural characteristics (*Ae. albopictus, Ae. aegypti, Culex quinquefasciatus, C. nigripalpus*) [46]. In Mayotte, this was observed between *Ae. albopictus* and *Ae. aegypti*; both species were capable of re-colonising the same larval development sites [47]. The fact that no temporal or container type clusters were observed in *Ae. albopictus* populations, suggests that there is no genetic adaptation to a particular type of larval habitat in this species, consistent with the observation that *Ae. albopictus* is thought to have broad ecological plasticity [11], [36].

No differentiation between vectorial competence for CHIKV was observed in populations from different localities in Réunion Island (not even between the eastern populations in Saint Benoît and the western populations in Saint Pierre) [8]. However, differences in human CHIKV infection rates were observed [24] which might probably due to the density of vectors.

### Conclusion

We have shown that urban areas are preferred by *Ae. albopictus* for mating and oviposition. This is likely due to host availability and the existence stable and abundant artificial containers that serve as larval development sites facilitating large mosquito densities. Gullies and other natural environments however, are potential reservoirs for *Ae. albopictus* on Réunion Island, for re-colonising the urban areas after a population reduction (for example, following vector control). Nevertheless, when available suitable larval development sites are abundant, low production of mosquitos and population structuring is observed. This suggests that females have a preference for certain habitats and reproductive isolation depending on the habitat. An important consequence of the existence of highly clustered, local spatial patterns is that if some houses are missed during vector control operations, it is possible that the remaining intact mosquito clusters could subsequently repopulate the area. These results underline the need to use new control methods as an alternative to chemical control, such as the sterile insect technique.

## Supporting Information

Figure S1 DK (Evanno *et al.* 2005) as obtained in Structure with Kmax ranging from 2–10. Each value was obtained by averaging the posterior probabilities of 10 independent runs.
(TIF)

Figure S2 Average co-ancestry coefficients in 12 populations of *Aedes albopictus* assigned to 2 clusters. Numbers and population codes according to Figure S1 and Table 1, respectively. Coefficients were obtained from the structure analysis illustrated in Figure S1 (see Materials and Methods section). The threshold for an individual belonging to population 1 or 2 was chosen as 0.70, below this level individuals were considered as hybrids (*i.e.* 0.3–0.7).
(TIF)
