# Supplementary figures and images for: Evidence of Habitat Structuring Aedes albopictus Populations in Réunion Island
Source: PLoS Negl Trop Dis. 2013 Mar 21;7(3):e2111. doi: 10.1371/journal.pntd.0002111 (PMC3605158; doi:10.1371/journal.pntd.0002111)

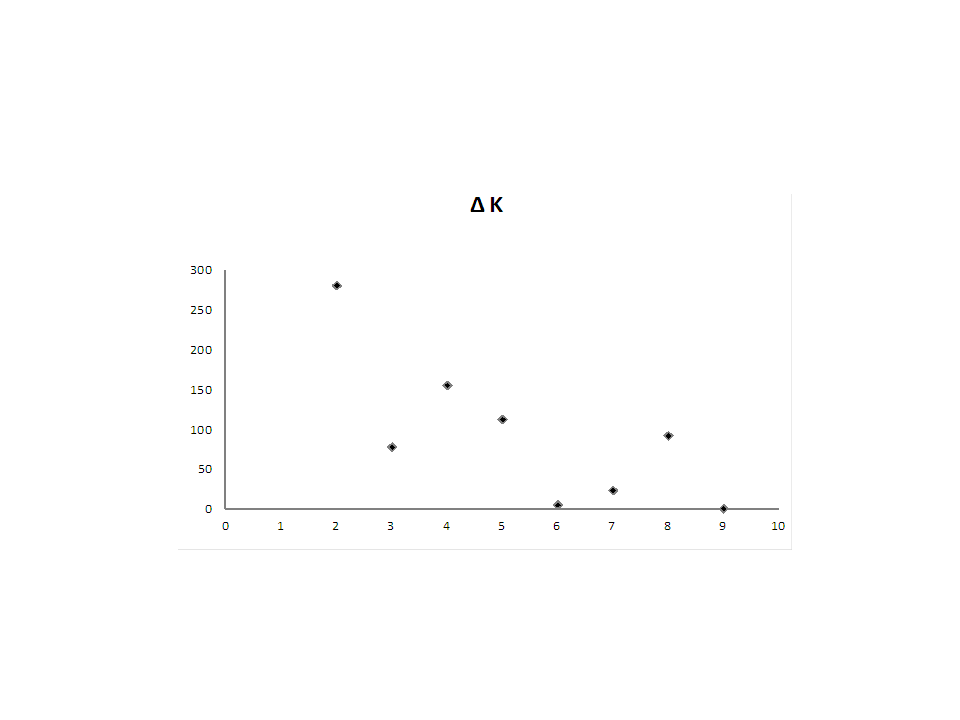

Supplement: Figure S1 — DK (Evanno et al. 2005) as obtained in Structure with Kmax ranging from 2–10. Each value was obtained by averaging the posterior probabilities of 10 independent runs. (TIF) [file pntd.0002111.s001.tif]

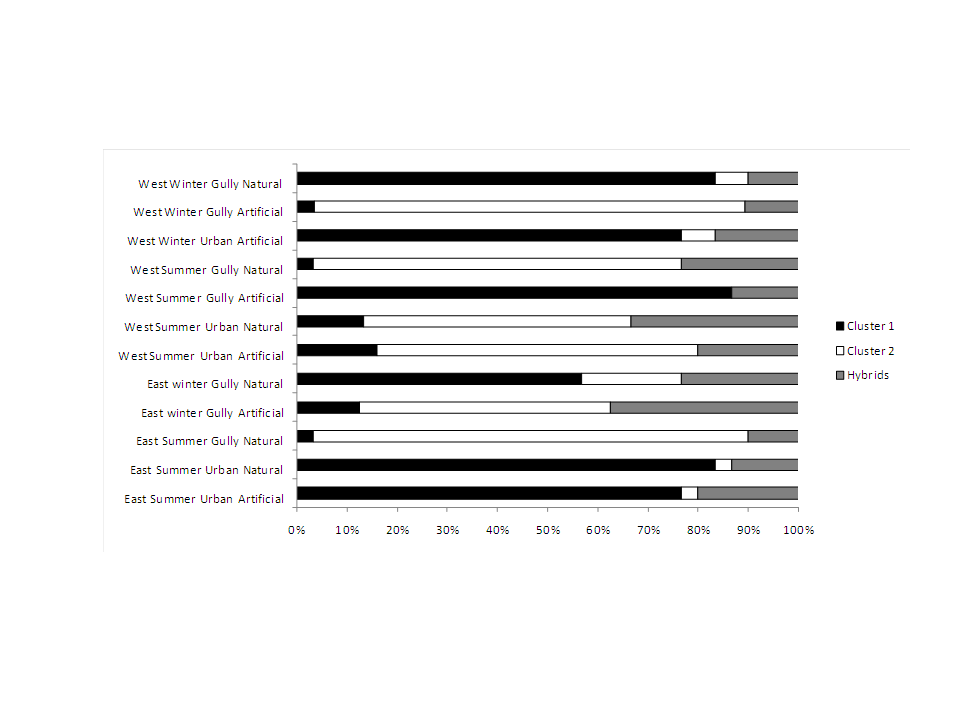

Supplement: Figure S2 — Average co-ancestry coefficients in 12 populations of Aedes albopictus assigned to 2 clusters. Numbers and population codes according to Figure S1 and Table 1, respectively. Coefficients were obtained from the structure analysis illustrated in Figure S1 (see Materials and Methods section). The threshold for an individual belonging to population 1 or 2 was chosen as 0.70, below this level individuals were considered as hybrids (i.e. 0.3–0.7). (TIF) [file pntd.0002111.s002.tif]
